# Albuminuria, serum creatinine, and estimated glomerular filtration rate as predictors of cardio-renal outcomes in patients with type 2 diabetes mellitus and kidney disease: a systematic literature review

**DOI:** 10.1186/s12882-018-0821-9

**Published:** 2018-02-09

**Authors:** Keith C. Norris, Karen E. Smoyer, Catherine Rolland, Jan Van der Vaart, Eliza Beth Grubb

**Affiliations:** 1 David Geffen School of Medicine at UCLA, Division of General Internal Medicine and Health Services Research, 911 Broxton Avenue, Room 103, Los Angeles, CA 90024 USA; 2 Envision Pharma Group, Philadelphia, PA USA; 3 Envision Pharma Group, Hammersmith, London, UK; 4 AbbVie, Hoofddorp, Haarlemmermeer The Netherlands; 5 AbbVie, Chicago, IL USA

**Keywords:** Albuminuria, Serum creatinine, Estimated glomerular filtration rate, Biomarker, Kidney disease progression, Type 2 diabetes mellitus

## Abstract

**Background:**

Albuminuria, elevated serum creatinine and low estimated glomerular filtration rate (eGFR) are pivotal indicators of kidney decline. Yet, it is uncertain if these and emerging biomarkers such as uric acid represent independent predictors of kidney disease progression or subsequent outcomes among individuals with type 2 diabetes mellitus (T2DM). This study systematically examined the available literature documenting the role of albuminuria, serum creatinine, eGFR, and uric acid in predicting kidney disease progression and cardio-renal outcomes in persons with T2DM.

**Methods:**

Embase, MEDLINE, and Cochrane Central Trials Register and Database of Systematic Reviews were searched for relevant studies from January 2000 through May 2016. PubMed was searched from 2013 until May 2016 to retrieve studies not yet indexed in the other databases. Observational cohort or non-randomized longitudinal studies relevant to albuminuria, serum creatinine, eGFR, uric acid and their association with kidney disease progression, non-fatal cardiovascular events, and all-cause mortality as outcomes in persons with T2DM, were eligible for inclusion. Two reviewers screened citations to ensure studies met inclusion criteria.

**Results:**

From 2249 citations screened, 81 studies were retained, of which 39 were omitted during the extraction phase (cross-sectional [*n* = 16]; no outcome/measure of interest [*n* = 13]; not T2DM specific [*n* = 7]; review article [*n* = 1]; editorial [*n* = 1]; not in English language [*n* = 1]). Of the remaining 42 longitudinal study publications, biomarker measurements were diverse, with seven different measures for eGFR and five different measures for albuminuria documented. Kidney disease progression differed substantially across 31 publications, with GFR loss (*n* = 9 [29.0%]) and doubling of serum creatinine (*n* = 5 [16.1%]) the most frequently reported outcome measures. Numerous publications presented risk estimates for albuminuria (*n* = 18), serum creatinine/eGFR (*n* = 13), or both combined (*n* = 6), with only one study reporting for uric acid. Most often, these biomarkers were associated with a greater risk of experiencing clinical outcomes.

**Conclusions:**

Despite the utility of albuminuria, serum creatinine, and eGFR as predictors of kidney disease progression, further efforts to harmonize biomarker measurements are needed given the disparate methodologies observed in this review. Such efforts would help better establish the clinical significance of these and other biomarkers of renal function and cardio-renal outcomes in persons with T2DM.

**Electronic supplementary material:**

The online version of this article (10.1186/s12882-018-0821-9) contains supplementary material, which is available to authorized users.

## Background

Chronic kidney disease (CKD) is a progressive condition characterized by a gradual decline in kidney function, which can result in end-stage renal disease (ESRD) [1]. The global prevalence of CKD is estimated to be between 8% and 16% [2], and is thought to exceed 50% in certain high-risk populations [3]. As of 2013, in the United States alone, more than 30 million adults were projected to have CKD, with the incidence rising profoundly among those aged 65 years and older [4].

The etiology of CKD is multifactorial, with glomerular hypertension and hyperfiltration reflecting the most prominent mechanistic contributors to disease progression [5]. Both systemic hypertension and glomerular hypertension resulting from glomerular hemodynamic changes, are known to provoke injury to the glomeruli. As a consequence, elevated blood pressure can overwhelm normal protection afforded from systemic hypertension to the kidney by autoregulation [5]. Hyperfiltration initiates the renin angiotensin aldosterone system (RAAS), which in turn, increases glomerular permeability and gives rise to albuminuria, proteinuria, and dyslipidemia, while also diminishing glomerular filtration rate (GFR), thus reciprocally leading to hypertension.

Concurrent with hypertension, which occurs in approximately 65% of the diabetic population [6], hyperglycemia and genetic predisposition are key factors in the development of kidney disease and in its progression [7]. Diabetic kidney disease (DKD) is typically characterized by persistent albuminuria, increasing serum creatinine and a progressive decline in estimated GFR (eGFR). Over time, worsening DKD is associated with increased risk of cardiovascular (CV) and cerebrovascular events, as well as renal morbidity and mortality [7]. Emerging data also suggest uric acid may be a potential marker of CKD [8]. Routine assessment of these key biomarkers is an important dimension of preventive medicine, as it helps to identify those who might benefit from earlier intervention to lower the risk of adverse outcomes.

Although increasing urinary albumin excretion and serum creatinine levels, along with diminished eGFR, are likely important markers of kidney decline, it is unclear whether these biomarkers reflect independent risk factors for kidney disease progression, subsequent outcomes in patients with type 2 diabetes mellitus (T2DM), or both. By extension, numerous pathophysiological mechanisms are implicated in DKD, and in this context, kidney disease progression in patients with T2DM could possibly be delayed or prevented by controlling other measures such as blood glucose or blood pressure levels [9–12, 7, 13]. Indeed, several randomized controlled trials have demonstrated that medications indicated for treatment of T2DM such as glucagon like peptide-1 (GLP-1) [14, 15] and sodium-glucose co-transporter 2 (SGLT-2) inhibitors [16, 17] can slow renal disease progression as well as reduce adverse CV outcomes. Other complementary therapeutic approaches such as utilizing selective endothelin receptor type A (ET-A) antagonists may also help delay renal function decline and subsequently lower the risk of related adverse renal outcomes [18]; albeit the precise beneficial mechanisms conferred from these medications are still undergoing investigation [19].

In light of these findings, a better understanding of the prognostic utility of DKD biomarkers and whether they might be implicated in increasing the risk of adverse renal outcomes in patients with T2DM, would help clarify their clinical value, and guide treatment decision-making in everyday clinical practice. The objective of this study was to conduct a systematic literature review (SLR) of the published evidence documenting the role of albuminuria, serum creatinine, eGFR, and uric acid in predicting kidney disease progression and cardio-renal outcomes among patients with T2DM.

## Methods

### Databases and search strategy

Embase, MEDLINE, and Cochrane Central Trials Register and Database of Systematic Reviews and other Cochrane Library assets were searched for human studies published in English between January 2000 and May 2016. Additionally, we performed a PubMed search from 2013 until May 2016, in an effort to capture more recently published studies that may not yet have been indexed in one of the other databases. Implementation and reporting of the present SLR adhered to the guidelines and standards advocated by the National Institute for Health and Care Excellence (NICE) and Preferred Reporting Items for Systematic Reviews and Meta-Analyses (PRISMA) guidelines [20]. Although review papers identified in the searches were omitted from this study, the reference lists for each identified review article were hand-screened to include any relevant referenced publications (*n* = 2) that met study inclusion criteria and had not been identified during the database searches.

The data sources above were searched to identify specific observational or non-randomized studies relevant to renal biomarkers and the need for renal replacement therapy. For the current SLR, we adopted the Population, Intervention, Comparator, Outcomes, and Study type (PICOS) criteria to establish our search strategy and inclusion/exclusion criteria. Relevant studies were subsequently identified using text word or MeSH headings, including but not confined to: population terms such as T2DM and non-insulin dependent diabetes; condition/disease outcome terms including renal or kidney replacement or transplantation, renal or kidney failure, chronic renal or kidney disease, end-stage renal or kidney disease; and biomarker/clinical measure terms including GFR, albuminuria, proteinuria, and creatinine.

Only full-text publications were included; congress abstracts were excluded. Studies of behavioral, educational, or clinical practice interventions were included. All review studies (expert, narrative, and systematic reviews, as well as meta-analyses), preclinical studies, pharmacovigilance/safety studies, case reports, and studies that employed a drug or device intervention were excluded from the SLR.

### Screening, critical appraisal, and quality assessment

At the first-level screening, one reviewer screened the titles of publications identified from the search according to the eligibility criteria. A second reviewer then performed a quality check of 10% of all screened publications. Where discrepancies existed in a screening decision among the 10% of publications, the second reviewer examined all of the publications that had been excluded based on the same reason as the study marked with the discrepancy. At the second-level screening, one reviewer screened the abstracts, and if necessary, full texts of publications retained from the first screening stage against the study eligibility criteria. A second reviewer then performed a quality check of 20% of all screened studies. In the event of a discrepancy, the second reviewer was tasked with checking all studies that had been excluded for the same reason as the study marked with the discrepancy. Any remaining discrepancies regarding study inclusion were resolved by a third reviewer.

Publications of longitudinal studies meeting eligibility criteria that described the role of albuminuria/proteinuria or serum creatinine/uric acid/eGFR in renal disease progression were included in this review. Although uric acid is a less well established potential biomarker of CKD risk, we examined it in this SLR with creatinine/eGFR. All studies meeting inclusion criteria were assessed for quality using the Downs and Black (D&B) instrument [21], which is suitable for non-randomized longitudinal clinical and observational studies. The D&B instrument evaluates (a) the methodological quality (10 items), (b) statistical power (one item), (c) bias (seven items), (d) confounding (six items), and (e) external validity of the study (three items).

### Study outcomes

The primary outcomes examined in this study were renal disease progression, ESRD, non-fatal CV events, and all-cause mortality. Renal disease progression reflected any mention of GFR loss, a steep decline in eGFR, at least a 50% decline in eGFR, a doubling in serum creatinine, or nephropathy progression. Non-fatal CV events comprised non-fatal myocardial infarction or non-fatal stroke.

### Statistical considerations

Key data were extracted from each of the selected publications, where available, and included: study type (e.g. retrospective or prospective); geographic region where the study was conducted; sample size; baseline patient demographics, clinical characteristics and reported medication use; clinical indicators and outcomes of kidney decline and disease progression; follow up duration; and time to renal mortality, CV mortality, all-cause mortality, or receipt of any renal replacement therapy (RRT) including chronic dialysis or transplantation. Descriptive reporting included tabulations of study design and patient characteristics. Risk estimates were extracted from the included publications. These primarily were hazard ratios, and in some cases relative risks (RR), with 95% confidence limits (where provided) for the association between renal biomarkers and outcomes of interest. Risk estimates reported in the publications were assessed qualitatively by visual inspection for statistical significance as well as directionality (e.g. an increase or reduction in the risk of an outcome of interest according to a particular biomarker). Due to the variation of study biomarker predictors, covariates, outcomes, statistical methods, and risk estimates used in the included publications, quantitative comparison of risk estimates across studies was not possible. Data extracted from the included publications were reported, tabulated, and descriptively assessed in Microsoft Excel 2016 worksheets (Microsoft Corporation, Redmond, WA, USA).

## Results

### Literature search and baseline data from included publications

**Fig. 1:**
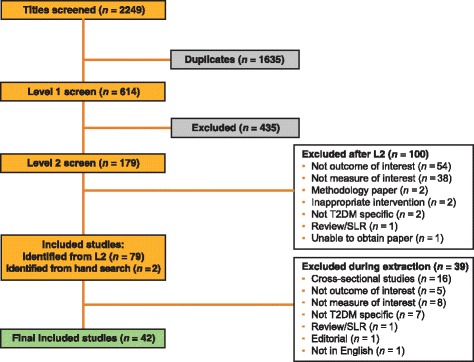
Preferred Reporting Items for Systematic Reviews and Meta-Analyses (PRISMA) flow chart *SLR* systematic literature review*, T2DM* type 2 diabetes mellitus

Database searches identified 2249 potential publications: 787 from Embase, 519 from MEDLINE, 74 from Cochrane Central Trials Register and Database of Systematic Reviews, and 868 from PubMed. After initial title screening and following deduplication, 614 publications remained, of which 179 were considered potentially suitable for inclusion. Of these, 42 publications met the relevant criteria to be included in this SLR (Fig. 1 and Table 1). The majority of studies included were prospective (*n* = 27; 64.3%), and varied in quality (i.e. out of a maximum of 27 points, mean score = 16.52; range = 12–20) as assessed using the D&B instrument (Table 1 and Additional file 1: Table 1).

**Table 1:** Summary of longitudinal publications meeting inclusion criteria and included in the systematic literature review

Author	Study name	Publication year	Overall sample size (n)	Sample size of smallest sub-group (n)	Mean follow-up (years)	Country	Overall quality assessment score (%)^a,b^
Afghahi et al. [22]	Swedish National Diabetes Register	2010	3667	407	5	Sweden	18 (67%)
Afkarian et al. [9]	NHANES III	2013	1430	658	10	USA	17 (63%)
Al Suleiman et al. [77]	N/A	2008	35	N/A	8	Saudi Arabia	16 (59%)
Altemtam et al. [23]	N/A	2011	270	94	5.2	UK	13 (48%)
Alwakeel et al. [44]	N/A	2011	621	166	10	Saudi Arabia	12 (44%)
Andresdottir et al. [10]	N/A	2014	543	286	5.7	Denmark	15 (56%)
Araki et al. [49]	Japanese Elderly Diabetes Intervention Trial	2012	621	306	4.3	Japan	15 (56%)
Azubike et al. [56]	N/A	2013	22	N/A	12	Nigeria	15 (56%)
Bentata et al. [24]	N/A	2014	144	26	4.1	Morocco	13 (48%)
Berhane et al. [25]	N/A	2011	2420	1503	10.2	USA	15 (56%)
Bruno et al. [26]	Casale Monferrato Study	2007	1538	21	11	Italy	19 (70%)
Chen et al. [27]	N/A	2012	487	65	6.6	Taiwan	16 (59%)
Cox et al. [28]	Diabetes Heart Study	2013	1220	N/A	8.2	USA	17 (63%)
De Cosmo et al. [8]	N/A	2015	13,964	2540	4	Italy	20 (74%)
de Hauteclocque et al. [37]	SURDIAGENE	2014	1146	486	5.7	France	19 (70%)
Dunkler et al. [29]	ONTARGET and ORIGIN	2015	15,066	6766	5.5	Multinational	15 (56%)
Elley et al. [38]	New Zealand Diabetes Cohort Study	2013	31,613	5877	7.3	New Zealand	16 (59%)
Jardine et al. [39]	ADVANCE	2012	11,140	7377	4.8	Multinational	19 (70%)
Kitai et al. [30]	N/A	2015	125	22	75 days	Japan	20 (74%)
Lambers Heerspink et al. [50]	RENAAL	2010	701	N/A	3.4	Multinational	16 (59%)
Monseu et al. [40]	SURDIAGENE	2015	1371	411	4.8	France	15 (56%)
Moriya et al. [54]	N/A	2012	30	9	6.2	Japan	14 (52%)
Murussi et al. [31]	N/A	2007	173	41	8	Brazil	17 (63%)
Packham et al. [11]	N/A	2012	3228	N/A	2.8	Multinational	19 (70%)
Pavkov et al. [12]	N/A	2008	983	N/A	8.4	USA	17 (63%)
Pavkov et al. [45]	N/A	2012	195	88	4	USA	15 (56%)
Pavkov et al. [41]	N/A	2013	234	76	10.7	USA	16 (59%)
Retnakaran et al. [13]	UKPDS	2006	9063	4031	15	UK	17 (63%)
Sinkeler et al. [42]	RENAAL and IDNT	2013	1872	623	3	Multinational	14 (52%)
Stoycheff et al. [52]	IDNT	2009	1608	693	2.6	Multinational	14 (52%)
Takagi et al. [46]	N/A	2015	1802	1655	6.9	Japan	18 (67%)
Tanaka et al. [47]	N/A	2015	3231	137	5.9	Japan	19 (70%)
Targher et al. [32]	Verona Diabetes Study	2011	2823	38	5.7	Italy	19 (70%)
Unsal et al. [33]	N/A	2012	122	35	3.3	Turkey	15 (56%)
Viana et al. [34]	N/A	2012	199	86	6.1	Brazil	15 (56%)
Vupputuri et al. [35]	N/A	2011	10,290	52	3.1	USA	17 (63%)
Wada et al. [48]	N/A	2013	4328	534	7	Japan	19 (70%)
Yang et al. [36]	Hong Kong Diabetes Registry	2006	4438	159	2.9	Hong Kong	19 (70%)
Yokoyama et al. [53]	JDDM	2011	2954	175	3.8	Japan	16 (59%)
Yokoyama et al. [55]	N/A	2012	211	28	4.5	Japan	18 (67%)
Yokoyama et al. [51]	N/A	2013	1002	303	3.8	Japan	16 (59%)
Zoppini et al. [43]	Verona Diabetes Study	2012	1682	263	10	Italy	19 (70%)

*NHANES III* Third National Health and Nutrition Examination Survey, *SURDIAGENE* Survie, Diabete de type 2 et Genetique Study, *ONTARGET* Ongoing Telmisartan Alone and in Combination with Ramipril Global Endpoint Trial, *ORIGIN* Outcome Reduction With Initial Glargine Intervention Trial, *ADVANCE* Action in Diabetes and Vascular Disease: Preterax and Diamicron MR Controlled Evaluation Study, *RENAAL* Reduction of Endpoints in NIDDM with the Angiotensin II Antagonist Losartan Study, *UKPDS* UK Prospective Diabetes Study, *IDNT* Irbesartan Diabetic Nephropathy Trial, *JDDM* Japan Diabetes Clinical Data Management Study

^a^Scored using the Downs and Black quality assessment instrument [21]

^b^Proportional score was calculated by dividing each study’s overall quality assessment score by the sum of points available in the Downs and Black instrument

**Fig. 2:**
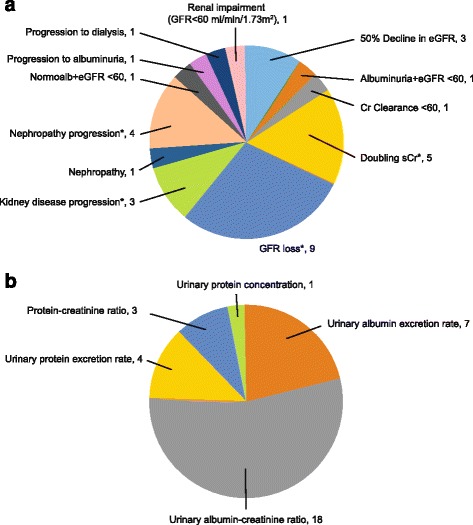
**a** Frequency of measures used to define kidney decline/chronic kidney disease progression in 31 longitudinal publications; (**b**) Frequency of measures used to define albuminuria/proteinuria in 33 longitudinal publications. *100% (3 out of 3) of 50% decline in eGFR, 60% (3 out of 5) of doubling of serum creatinine, and 33% (1 out of 3) of kidney disease progression measures included the composite end points of renal replacement therapy initiation, end-stage renal disease, or mortality. Nephropathy progression did not include composite end points, instead, three studies reported “nephropathy progression” and one reported “worsening of nephropathy stage”. *CKD* chronic kidney disease, *Cr* creatinine, *eGFR* estimated glomerular filtration rate, *GFR* glomerular filtration rate, *s* serum

**Fig. 3:**
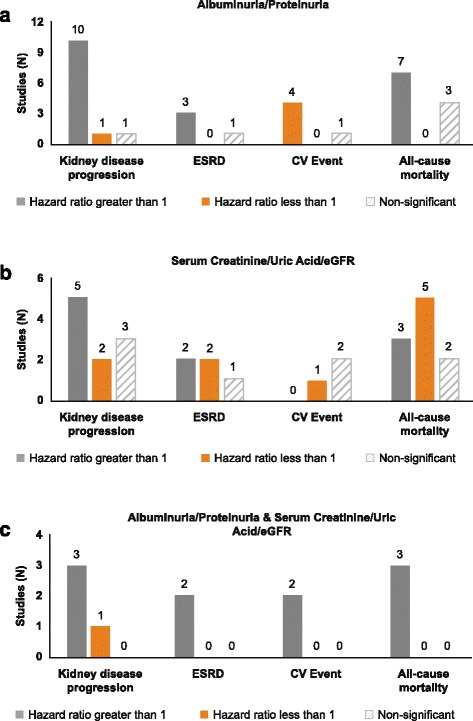
Number of publications reporting a significant (direct or inverse) or non-significant relationship for risk estimates with clinical outcomes according to the biomarkers albuminuria/proteinuria, serum creatinine/uric acid/eGFR, or both measured simultaneously, in patients with type 2 diabetes mellitus. *eGFR* estimated glomerular filtration rate, *ESRD* end-stage renal disease, *CV* cardiovascular. Panel **a**. Hazard ratios for studies with albuminuria/proteinuria outcomes. Panel **b**. Hazard ratios for studies with serum creatinine/uric acid/eGFR outcomes. Panel **c**. Hazard ratios for studies with albuminuria/proteinuria and serum creatinine/uric acid/eGFR

Baseline CKD stage was reported in fewer than 5% of included publications (Table 2). To estimate patient severity, CKD stage was inferred from eGFR, where available. An extensive range of baseline characteristics was observed, encompassing patients with normal to severely impaired kidney function (Table 2). Seven different measures of eGFR were reported, with the modification of diet in renal disease (MDRD; *n* = 16; 43.2%) [22–31, 11, 32–36] and the Chronic Kidney Disease Epidemiology Collaboration (CKD-EPI; *n* = 10; 27.0%) [9, 25, 37, 29, 38–43] the most frequently used (Table 2). Measures of kidney decline/disease progression were diverse. After removing composite end points, 12 different measures were reported across 31 publications (Fig. 2a). GFR loss (*n* = 9; 29.0%) [23, 44, 10, 37, 30, 45–48] and doubling of serum creatinine (*n* = 5; 16.1%) [49, 50, 40, 13, 51] were the most commonly reported measures of kidney disease progression in the included longitudinal studies (Fig. 2a). Measures of albuminuria and proteinuria also varied, with five different measures reported among 33 publications (Fig. 2b). Of these, the most frequently reported markers were urinary albumin-to-creatinine ratio (*n* = 18 publications; 54.5%) [49, 28, 37, 29, 39, 50, 40, 11, 12, 41, 45, 52, 46–48, 36, 51, 53] and urinary albumin excretion rate (*n* = 7; 21.2%) [10, 26, 50, 54, 31, 42, 52]. A summary of findings from publications providing risk estimates for outcomes of interest by each of the investigated biomarkers is presented in Fig. 3. Biomarkers including baseline albuminuria/proteinuria, serum creatinine/uric acid/eGFR, or both combined, were most often associated with an increased risk of kidney disease progression and all-cause mortality, with more papers reporting a significant rather than a non-significant relationship (Fig. 3).

**Table 2:** Baseline characteristics derived from longitudinal study populations

Characteristic	Studies reporting characteristic, *N* (%)	Category	*n* (%) or range
Study type	42 (100%)	Prospective Retrospective Predictive model Registry	27 (64.3%) 10 (23.8%) 3 (7.1%) 2 (4.8%)
Age (years)	42 (100%)	–	29–100
Race/ethnicity^a^	30 (71.4%)	Caucasian Black Asian Other	13 (43.3%) 10 (33.3%) 11 (36.7%) 11 (36.7%)
CKD stage determination	42 (100%)	CKD stage reported CKD stage inferred CKD undetermined	2 (4.8%) 36 (85.7%) 4 (9.5%)
BMI (kg/m^2^)	40 (95.2%)	–	16.5–44.9
Diabetes duration (years)	35 (83.3%)	–	1–40
HbA1c (%)	39 (92.9%)	–	5.4–11.9%
Systolic BP (mm Hg)	41 (97.6%)	–	100–182
Diastolic BP (mm Hg)	37 (88.1%)	–	53–130
eGFR (ml/min/1.73m^2^)	38 (90.5%)	–	10–228
eGFR assessment method^b^	37 (88.1%)	MDRD CKD-EPI JSN Cockcroft-Gault 51 Cr-EDTA Iohexal clearance Iothalamide clearance	16 (43.2%) 10 (27.0%) 6 (16.2%) 3 (8.1%) 2 (5.4%) 1 (2.7%) 1 (2.7%)
ACR (mg/gCR)	22 (52.4%)	–	0.5–8603
sUA (mg/dL)	3 (7.1%)	–	4.9–7.4

*CKD* chronic kidney disease, *BMI* body mass index, *HbA1c* hemoglobin A1c, *BP* blood pressure, *eGFR* estimated glomerular filtration rate, *MDRD* modification of diet in renal disease, *CKD-EPI* chronic kidney disease epidemiology, *JSN* Japanese society of nephrology, *ACR* albuminuria-to-creatinine ratio, *sUA* serum uric acid

^a^More than one type of race/ethnicity was reported in multiple studies

^b^More than one assessment method was obtained in two studies

### Biomarkers as independent predictors of clinical outcomes

#### Albuminuria/proteinuria

Twenty-two publications presented data on albuminuria/proteinuria [23, 44, 10, 25–28, 37, 38, 30, 50, 40, 31, 45–47, 32, 34, 48, 36, 55, 51], of which 18 provided risk estimates for albuminuria/proteinuria as a biomarker for an outcome of interest (Additional file 1: Table S2). Albuminuria was measured at baseline as well as follow up, both by absolute measure and in terms of doubling from baseline. The included studies documented associations between albuminuria and a wide range of renal outcomes. For instance, albuminuria was identified as a significant risk factor in kidney disease progression, as determined by reduced GFR (*n* = 5) [37, 45–48]; a steep rate of decline in GFR (where albuminuria was elevated, *n* = 2) [37, 30]; and a doubling of serum creatinine (*n* = 2) [50, 40] (Additional file 1: Table S2). One publication also documented a reduced risk of doubling of serum creatinine accompanying macro-albuminuria remission [51]. In four publications, both increased baseline albuminuria as well as elevated levels of albuminuria over time were significantly associated with time to ESRD [25, 37, 45, 36]. However, one of these studies reported no measures of precision (confidence intervals) or test statistics (*p*-value) [25]; hence, statistical significance could not be ascertained (Additional file 1: Table S2). A separate publication reported a greater risk for ESRD in relation to micro-, macro-, and advanced albuminuria [38]. Four publications reported baseline micro- and macro-albuminuria as significant predictors of experiencing a CV event [40, 34, 48, 55]. For all-cause mortality, seven publications reported a larger risk observed on the background of higher baseline levels of albuminuria [26, 28, 40, 31, 32, 34, 48] (Additional file 1: Table S2).

#### Serum creatinine/urine creatinine/uric acid/eGFR

Twenty three publications provided data on baseline serum creatinine, uric acid, and eGFR as risk factors [23, 44, 10, 49, 56, 24–28, 37, 38, 50, 40, 31, 45, 13, 42, 46, 47, 32, 36, 55], with 13 reporting the relationship between serum creatinine, uric acid, or eGFR with an outcome of interest (Additional file 1: Table S3). One study found that a higher baseline eGFR was protective against GFR decline [46]. Another reported that an increase in serum uric acid was associated with a greater risk of doubling of serum creatinine [49]. Similarly, a decrement of 1 standard deviation in 24-h urinary creatinine clearance and 24-h urinary creatinine excretion significantly increased the risk of doubling of serum creatinine [50]. Conversely, the risk of doubling of serum creatinine was significantly lower with higher baseline eGFR levels indicative of better kidney function [40], and a higher baseline eGFR was also associated with a lower risk of ESRD [36]. A lower baseline eGFR or steep decline in eGFR over time were both significantly associated with an earlier onset of ESRD [37, 45] (Additional file 1: Table S3). Available data for time to experiencing a CV event were sparse for these biomarkers, and in some instances, conflicting. For example, non-significant associations between baseline eGFR and time to experiencing a CV event were documented in two publications [27, 55], whereas in another, a significant reduction in the risk of experiencing a CV event was observed [40] (Additional file 1: Table S3). For all-cause mortality, significant predictors included increasing serum creatinine [28] and decreasing eGFR [32]. In addition, higher eGFR concentrations [28, 40, 47] and a higher creatinine excretion rate [42] were associated with improved survival (Additional file 1: Table S3).

#### Albuminuria/proteinuria and serum creatinine/uric acid/eGFR

Six publications reported data for albuminuria/proteinuria and serum creatinine/uric acid/eGFR measured in combination [25, 8, 11, 35, 48, 55], of which all documented risk estimates for these combined measures with an outcome of interest (Additional file 1: Table S4). Normo-albuminuria in the presence of an eGFR < 30 ml/min/1.73m^2^ was associated with kidney disease progression [48], shorter time to ESRD [25, 11], and shorter time to experiencing a CV event [48]. One publication documented an increase in risk of kidney disease progression according to higher serum uric acid on the background of normoalbuminuria and an eGFR < 60 ml/min/1.73m^2^ [7]. Another publication reported a significant increase in risk of kidney disease progression according to change in eGFR among patients with normo-albuminuria, but also reported a significant reduction in risk of kidney disease progression according to changes in eGFR in patients with baseline macro-albuminuria [35]. The latter finding may be explained, in part, by the higher number of patients who might have attained a regression in albuminuria within the macro-albuminuria group. Micro- and macro-albuminuria at baseline were significantly associated with progression of kidney disease [48], a shorter time to ESRD [25, 11] or to a CV event [48, 55], regardless of eGFR status (Additional file 1: Table S4). A trend towards increased albuminuria, but not eGFR decline, was significantly associated with shorter time to a CV event as well as to all-cause mortality [55]. In other studies, time to all-cause mortality was also shorter among patients with baseline micro- [25] and macro- [25, 48, 55] albuminuria. These latter studies did not examine eGFR decline but rather baseline eGFR and similarly found it was not a significant predictor (Additional file 1: Table S4).

## Discussion

This study aimed to systematically review the published literature regarding the role of the biomarkers albuminuria, serum creatinine, eGFR, and uric acid in predicting kidney disease progression and associated cardio-renal outcomes in persons with T2DM. Of the 42 longitudinal publications identified in this review, data reported for the baseline population, biomarkers, and outcomes investigated were for the large part heterogeneous. Biomarker measures tended to be dissimilar between studies, with multiple measures employed to assess eGFR and albuminuria. Further contributing to study heterogeneity, 12 different outcome measures were identified for kidney decline or disease progression alone, which made cross-study comparisons a challenge.

Irrespective of these discrepancies, the data reviewed in this study showed that albuminuria, serum creatinine/eGFR, or the combination of both, were robust predictors of adverse outcomes in persons with T2DM. Of the three biomarkers, albuminuria was the most frequently evaluated, with the majority of studies identified in this review displaying an association with kidney decline and related outcomes, which is fitting with prior investigations [57, 31, 58]. Additional evidence in patients with T2DM has indicated that baseline micro- and macro-albuminuria as well as increasing albuminuria carry higher risks of declining kidney function and associated outcomes, beyond other existing renal biomarkers [59]. In one meta-analysis of five studies encompassing patients with T2DM, patients with micro-albuminuria displayed an almost 4-fold (95% CI 1.6–8.4) increased RR for developing ESRD as compared with those who had normo-albuminuria [60]. Absolute changes in albuminuria were also considered as precursors to kidney disease progression in individuals with T2DM [61], with ample data further emphasizing the relationship between albuminuria and incipient nephropathy as well as CV outcomes [62, 63]. Taken together, these findings support the utility of albuminuria as a robust predictor of renal decline. Hence, routine screening for albuminuria in persons with T2DM is considered effective clinical practice for monitoring the onset and progression of kidney disease.

Nevertheless, in light of the extant epidemiologic evidence, the clinical importance of albuminuria as a prognosticator of kidney disease has recently been challenged [64, 61, 65]. Although albuminuria is commonly used to assess kidney disease development and progression among patients with T2DM, high variability has been observed in this individual marker along with insufficient sensitivity or specificity to detect kidney disease end points on its own [66, 61, 67, 68]. By extension, the predictive utility of micro-albuminuria has further come into question due to observed spontaneous remission in people with diabetes [69, 61, 70]. On this basis, seeking out novel biomarkers that can more reliably identify individuals at risk of experiencing poor renal outcomes is warranted. Yet to date, most studies have only assessed the relationship of individual, rather than combined or composite biomarkers, with renal outcomes [71], and no new single biomarker has been shown capable of outperforming albuminuria [72]. Given the numerous pathophysiological processes that encompass DKD (e.g. hyperfiltration, pro-inflammatory, pro-fibrotic, and angiogenic processes) [73], it is doubtful that albuminuria, or any other single biomarker, is individually capable of accurately forecasting the development and progression of renal damage in persons with T2DM [68]. On the other hand, a more comprehensive selection of multiple biomarkers that capture distinct pathways involved in renal impairment would presumably augment risk prediction more effectively than the use of a single biomarker alone. Indeed, extending our efforts in this study beyond the conventional “albuminuric pathway” has been described elsewhere [66, 61, 67, 71, 68], and provides impetus for others to establish a panel of biomarkers intended to improve early detection of kidney disease development, as well as its progression in diabetes. For effective clinical use, this panel would need to be highly sensitive and specific, minimally invasive to collect, and cost-effective.

Several publications identified in this review demonstrated that patients with T2DM may present with low eGFR yet be normo-albuminuric. In this context, normo-albuminuric low eGFR has been reported to be more prevalent among older women [67], with these patients often exhibiting a more diverse set of clinical characteristics compared with those who present with low eGFR and higher concentrations of albuminuria [22, 74, 75]. Additionally, renal biomarkers are known to be influenced by clinical features such as age, gender, and genetic disparities, as well as modifiable factors [61, 76]. To this end, distinct sets of risk factors have been confirmed on the background of low eGFR, and separately for increased albuminuria [13], making it challenging to disentangle whether changes in these biomarkers are complementary of other underlying pathophysiological conditions not linked to kidney disease, or whether they truly reflect important markers of kidney disease development and progression in T2DM. Moving forward, other avenues of research appear warranted that will focus on assessing the predictive value of albuminuria, eGFR and other renal markers for kidney disease end points according to relevant subsets of conventional risk factors (i.e. age, race, or sex-specific disparities) in people with T2DM.

The findings from this SLR illustrated that the risk of kidney function decline and related outcomes tended to vary depending on which biomarker was studied. The available findings demonstrated that the risk as well as the onset of kidney function decline appeared to occur sooner in patients with T2DM who demonstrated an increase in albuminuria, or in the presence of a diminished eGFR over time, and that use of the two biomarkers combined had better predictive ability over time than either alone; although data were sparse. For this reason, studies incorporating these biomarkers in tandem, and their relationship with cardio-renal outcomes, would likely enrich our understanding of the numerous pathways involved in provoking renal impairment, especially given the high individual variability that often accompanies these biomarkers. To further improve prediction based on these studied biomarkers, standardized measures are also needed, as well as more reliable reporting of baseline kidney function and outcomes, particularly for kidney disease progression. Such standardizations will facilitate understanding of the clinical utility of albuminuria and serum creatinine as predictive biomarkers for kidney disease progression as well as related renal and CV outcomes and mortality risk among patients with T2DM, with the risk perhaps differing among select subgroups.

### Limitations

The observed heterogeneity in the published data clearly limits the generalizability of our findings regarding the importance of albuminuria, serum creatinine, and eGFR as biomarkers for adverse renal outcomes. Although a rigorous, objective, and transparent systematic methodology was implemented and quantitative data were extracted from the included publications, the present SLR was descriptive and qualitative in nature due to the widespread disparity observed among the study designs, baseline population characteristics, biomarkers measured, outcomes reported, and study methods and reporting employed. The majority of included publications did not clearly report baseline CKD stage for the study populations. We therefore referred to eGFR, where available, to estimate baseline kidney function; and it should be noted that these estimates were based on heterogeneous data, as numerous methods were used to define eGFR. The evaluated publications, despite meeting inclusion criteria for this SLR, did not report outcomes consistently, particularly for kidney disease progression, which made it difficult to compare predictors across the studies examined. The scientific quality of the data presented varied; for example, some studies had small sample sizes and not all publications reported risk estimates such as hazard ratios or measures of precision. Others failed to report a test statistic for significance. Of the studies that did report hazard ratios, the range of covariates included in survival models also varied, further making it challenging to directly compare risk estimates across each investigation.

## Conclusion

From the literature reviewed, albuminuria, serum creatinine, and eGFR were identified as the major potential predictors for the risk of kidney disease progression in patients with T2DM, with uric acid considered in a subset of articles. These biomarkers displayed some prediction towards kidney disease outcomes, and the few publications that assessed these biomarkers in tandem found additional predictive value for kidney disease progression not apparent for any of the biomarkers alone. Further efforts are needed to improve our understanding of the roles these markers of renal function might play in terms of risk prediction, in light of the measurement and methodologic disparities observed across the extant literature. As the global prevalence of CKD increases, particularly among individuals with T2DM, establishing a more reliable and consistent approach to measuring routine clinical parameters of renal function, along with a more standardized means for defining related outcomes, will help guide clinical decision-making and treatment initiatives designed to mitigate the ever-growing burden of renal disease.

## Additional files

Description of data The data provided in these additional files are in support of displaying more granular information for describing the individual quality assessment scores based on the Downs and Black quality assessment instrument, as well as the risk estimates according to each of the biomarkers examined in the longitudinal publications.

Additional file 1: Table S1. Individual quality assessment scores for each publication according to the Downs & Black quality assessment tool. **Table S2.** Risk estimates for albuminuria/proteinuria measures according to clinical outcomes reported in longitudinal publications. **Table S3.** Risk estimates for serum creatinine, uric acid, and estimated glomerular filtration rate measures according to clinical outcomes reported in longitudinal publications. **Table S4.** Risk estimates for combined albuminuria/proteinuria and serum creatinine/uric acid/estimated glomerular filtration rate measures according to clinical outcomes reported in longitudinal publications. (DOCX 315 kb)

## Abbreviations

ACR: Albuminuria-to-creatinine ratio
ADVANCE: Action in diabetes and vascular disease: preterax and diamicron MR controlled evaluation study
AER: Albumin excretion rate
BMI: Body mass index
BP: Blood pressure
CI: Confidence interval
CKD: Chronic kidney disease
CKD-EPI: Chronic kidney disease epidemiology
Cr: Creatinine
CV: Cardiovascular
D & B: Downs and black
DKD: Diabetic kidney disease
eGFR: Estimated glomerular filtration rate
ESRD: End-stage renal disease
ET-A: Endothelin receptor type A
FMV: First morning void
GFR: Glomerular filtration rate
GLP-1: Glucagon like peptide-1
HbA1c: Hemoglobin A1c
HR: Hazard ratio
IDNT: Irbesartan Diabetic Nephropathy Trial
JDDM: Japan diabetes clinical data management study
JSN: Japanese society of nephrology
MACE: Major adverse coronary event
MDRD: Modification of diet in renal disease
NHANES III: Third National Health and Nutrition Examination Survey
NICE: National Institute for Health and Care Excellence
ONTARGET: Ongoing Telmisartan Alone and in Combination with Ramipril Global Endpoint Trial
ORIGIN: Outcome reduction with initial glargine intervention trial
PAR: Peripheral artery revascularization
PICOS: Population, intervention, comparator, outcomes, and study type
PRISMA: Preferred reporting items for systematic reviews and meta-analyses
RAAS: Renin angiotensin aldosterone system
RENAAL: Reduction of Endpoints in NIDDM with the Angiotensin II Antagonist Losartan Study
RR: Relative risk
RRT: Renal replacement therapy
S: Serum
SD: Standard deviation
SGLT-2: Sodium-glucose co-transporter 2
SLR: Systematic literature review
sUA: Serum uric acid
SURDIAGENE: Survie, Diabete de type 2 et Genetique Study
T2DM: Type 2 diabetes mellitus
UAC: Urinary albumin concentration
UACR: Urinary albumin-to-creatinine ratio
UAE: Urinary albumin excretion
UCC: Urinary creatinine concentration
UCE: Urinary creatinine excretion
UKPDS: UK prospective diabetes study
UPE: Urinary protein excretion
