# Supplementary material for: Albuminuria, serum creatinine, and estimated glomerular filtration rate as predictors of cardio-renal outcomes in patients with type 2 diabetes mellitus and kidney disease: a systematic literature review
Source: BMC Nephrol. 2018 Feb 9;19:36. doi: 10.1186/s12882-018-0821-9 (PMC5807748; doi:10.1186/s12882-018-0821-9)
Supplement: Supplementary file 1 — Individual quality assessment scores for each publication according to the Downs & Black quality assessment tool. Table S2. Risk estimates for albuminuria/proteinuria measures according to clinical outcomes reported in longitudinal publications. Table S3. Risk estimates for serum creatinine, uric acid, and estimated glomerular filtration rate measures according to clinical outcomes reported in longitudinal publications. Table S4. Risk estimates for combined albuminuria/proteinuria and serum creatinine/uric acid/estimated glomerular filtration rate measures according to clinical outcomes reported in longitudinal publications. (DOCX 315 kb) [file 12882_2018_821_MOESM1_ESM.docx]

**Additional Files**

| **Additional file: Table 1** Individual quality assessment scores for each publication according to the Downs & Black quality assessment tool | | | | | | | | | | | | | | | | | | | | | | | | | | | | |
| --- | --- | --- | --- | --- | --- | --- | --- | --- | --- | --- | --- | --- | --- | --- | --- | --- | --- | --- | --- | --- | --- | --- | --- | --- | --- | --- | --- | --- |
|  | **Reporting** | | | | | | | | | | **External validity** | | | **Internal validity bias** | | | | | | | **Internal validity confounding** | | | | | | | **Overall quality assessment score (%)^*¶^** |
| **QA assessment number** | **1** | **2** | **3** | **4** | **5** | **6** | **7** | **8** | **9** | **10** | **11** | **12** | **13** | **14** | **15** | **16** | **17** | **18** | **19** | **20** | **21** | **22** | **23** | **24** | **25** | **26** | **27** | - |
| **Author** |  |  |  |  |  |  |  |  |  |  |  |  |  |  |  |  |  |  |  |  |  |  |  |  |  |  |  |  |
| Afghahi et al. [[1](#_ENREF_1)] | 1 | 1 | 1 | 0 | 2 | 1 | 1 | 0 | 0 | 1 | 0 | 1 | 1 | 0 | 0 | 1 | 1 | 1 | 0 | 1 | 1 | 1 | 0 | 0 | 1 | 1 | 0 | 18 (67%) |
| Afkarian et al. [[2](#_ENREF_2)] | 1 | 1 | 1 | 0 | 2 | 1 | 1 | 0 | 0 | 1 | 1 | 1 | 0 | 0 | 0 | 1 | 1 | 1 | 0 | 1 | 1 | 1 | 0 | 0 | 1 | 0 | 0 | 17 (63%) |
| Al Suleiman et al. [[3](#_ENREF_3)] | 1 | 1 | 1 | 0 | 1 | 1 | 1 | 0 | 0 | 1 | 1 | 1 | 1 | 0 | 0 | 1 | 1 | 1 | 0 | 1 | 1 | 1 | 0 | 0 | 0 | 0 | 0 | 16 (59%) |
| Altemtam et al. [[4](#_ENREF_4)] | 1 | 1 | 1 | 0 | 1 | 1 | 1 | 0 | 0 | 1 | 0 | 0 | 1 | 0 | 0 | 1 | 0 | 1 | 0 | 1 | 1 | 1 | 0 | 0 | 0 | 0 | 0 | 13 (48%) |
| Alwakeel et al. [[5](#_ENREF_5)] | 1 | 1 | 1 | 0 | 1 | 1 | 1 | 0 | 0 | 1 | 0 | 0 | 0 | 0 | 0 | 1 | 0 | 1 | 0 | 1 | 1 | 1 | 0 | 0 | 0 | 0 | 0 | 12 (44%) |
| Andresdottir et al. [[6](#_ENREF_6)] | 1 | 1 | 1 | 0 | 2 | 1 | 1 | 0 | 0 | 1 | 1 | 0 | 0 | 0 | 0 | 1 | 0 | 1 | 0 | 1 | 1 | 1 | 0 | 0 | 1 | 0 | 0 | 15 (56%) |
| Araki et al. [[7](#_ENREF_7)] | 1 | 1 | 1 | 0 | 1 | 1 | 1 | 0 | 0 | 1 | 1 | 0 | 1 | 0 | 0 | 1 | 0 | 1 | 0 | 1 | 1 | 1 | 1 | 0 | 0 | 0 | 0 | 15 (56%) |
| Azubike et al. [[8](#_ENREF_8)] | 0 | 1 | 1 | 1 | 1 | 1 | 1 | 0 | 1 | 1 | 0 | 0 | 0 | 0 | 0 | 1 | 1 | 1 | 0 | 1 | 1 | 1 | 0 | 0 | 0 | 1 | 0 | 15 (56%) |
| Bentata et al. [[9](#_ENREF_9)] | 1 | 1 | 1 | 0 | 1 | 1 | 1 | 0 | 1 | 1 | 0 | 0 | 0 | 0 | 0 | 1 | 0 | 1 | 0 | 1 | 1 | 0 | 0 | 0 | 1 | 0 | 0 | 13 (48%) |
| Berhane et al. [[10](#_ENREF_10)] | 1 | 1 | 1 | 0 | 1 | 1 | 1 | 0 | 0 | 0 | 1 | 1 | 0 | 0 | 0 | 1 | 1 | 1 | 0 | 1 | 1 | 1 | 0 | 0 | 1 | 0 | 0 | 15 (56%) |
| Bruno et al. [[11](#_ENREF_11)] | 1 | 1 | 1 | 0 | 2 | 1 | 1 | 0 | 1 | 1 | 1 | 1 | 0 | 0 | 0 | 1 | 1 | 1 | 0 | 1 | 1 | 1 | 0 | 0 | 1 | 1 | 0 | 19 (70%) |
| Chen et al. [[12](#_ENREF_12)] | 1 | 1 | 1 | 0 | 2 | 1 | 1 | 0 | 0 | 0 | 1 | 1 | 0 | 0 | 0 | 1 | 0 | 1 | 0 | 1 | 1 | 1 | 0 | 0 | 1 | 1 | 0 | 16 (59%) |
| Cox et al. [[13](#_ENREF_13)] | 1 | 1 | 1 | 0 | 2 | 1 | 1 | 0 | 0 | 1 | 1 | 1 | 0 | 0 | 0 | 1 | 0 | 1 | 0 | 1 | 1 | 1 | 0 | 0 | 1 | 1 | 0 | 17 (63%) |
| De Cosmo et al. [[14](#_ENREF_14)] | 1 | 1 | 1 | 0 | 2 | 1 | 1 | 0 | 1 | 1 | 1 | 1 | 1 | 0 | 0 | 1 | 1 | 1 | 0 | 1 | 1 | 1 | 0 | 0 | 1 | 1 | 0 | 20 (74%) |
| de Hauteclocque et al. [[15](#_ENREF_15)] | 1 | 1 | 1 | 0 | 2 | 1 | 1 | 0 | 0 | 1 | 1 | 1 | 1 | 0 | 0 | 1 | 0 | 1 | 0 | 1 | 1 | 1 | 1 | 0 | 1 | 1 | 0 | 19 (70%) |
| Dunkler et al. [[16](#_ENREF_16)] | 1 | 1 | 1 | 0 | 2 | 1 | 0 | 0 | 0 | 0 | 1 | 0 | 0 | 0 | 0 | 1 | 1 | 1 | 0 | 1 | 1 | 0 | 1 | 0 | 1 | 1 | 0 | 15 (56%) |
| Elley et al. [[17](#_ENREF_17)] | 1 | 1 | 1 | 0 | 2 | 1 | 0 | 0 | 0 | 0 | 1 | 1 | 1 | 0 | 0 | 1 | 0 | 1 | 0 | 1 | 1 | 1 | 0 | 0 | 1 | 1 | 0 | 16 (59%) |
| Jardine et al. [[18](#_ENREF_18)] | 1 | 1 | 1 | 0 | 2 | 1 | 1 | 0 | 0 | 1 | 1 | 1 | 0 | 0 | 0 | 1 | 1 | 1 | 0 | 1 | 1 | 1 | 1 | 0 | 1 | 1 | 0 | 19 (70%) |
| Kitai et al. [[19](#_ENREF_19)] | 1 | 1 | 1 | 0 | 2 | 1 | 1 | 0 | 1 | 1 | 1 | 1 | 1 | 0 | 0 | 1 | 1 | 1 | 0 | 1 | 1 | 1 | 0 | 0 | 1 | 1 | 0 | 20 (74%) |
| Lambers Heerspink et al. [[20](#_ENREF_20)] | 1 | 1 | 1 | 0 | 2 | 1 | 1 | 0 | 1 | 0 | 0 | 0 | 0 | 1 | 1 | 1 | 0 | 1 | 0 | 1 | 0 | 0 | 1 | 0 | 1 | 1 | 0 | 16 (59%) |
| Monseu et al. [[21](#_ENREF_21)] | 1 | 1 | 1 | 0 | 2 | 1 | 1 | 0 | 0 | 1 | 0 | 0 | 0 | 0 | 0 | 1 | 0 | 1 | 0 | 1 | 1 | 1 | 0 | 0 | 1 | 1 | 0 | 15 (56%) |
| Moriya et al. [[22](#_ENREF_22)] | 1 | 1 | 1 | 0 | 2 | 1 | 1 | 0 | 1 | 1 | 0 | 0 | 0 | 0 | 0 | 1 | 0 | 1 | 0 | 1 | 1 | 0 | 0 | 0 | 0 | 1 | 0 | 14 (52%) |
| Murussi et al. [[23](#_ENREF_23)] | 1 | 1 | 1 | 0 | 2 | 1 | 1 | 0 | 0 | 1 | 0 | 0 | 1 | 0 | 0 | 1 | 1 | 1 | 0 | 1 | 1 | 1 | 0 | 0 | 1 | 1 | 0 | 17 (63%) |
| Packham et al. [[24](#_ENREF_24)] | 1 | 1 | 1 | 0 | 2 | 1 | 1 | 0 | 1 | 1 | 1 | 1 | 0 | 1 | 1 | 1 | 0 | 1 | 0 | 1 | 1 | 0 | 1 | 0 | 1 | 0 | 0 | 19 (70%) |
| Pavkov et al. [[25](#_ENREF_25)] | 1 | 1 | 1 | 0 | 2 | 1 | 1 | 0 | 0 | 1 | 1 | 1 | 0 | 0 | 0 | 1 | 0 | 1 | 0 | 1 | 1 | 1 | 0 | 0 | 1 | 1 | 0 | 17 (63%) |
| Pavkov et al. [[26](#_ENREF_26)] | 1 | 1 | 1 | 0 | 2 | 1 | 1 | 0 | 0 | 1 | 1 | 1 | 0 | 0 | 0 | 1 | 0 | 1 | 0 | 1 | 1 | 0 | 0 | 0 | 1 | 0 | 0 | 15 (56%) |
| Pavkov et al. [[27](#_ENREF_27)] | 1 | 1 | 1 | 0 | 2 | 1 | 1 | 0 | 0 | 1 | 1 | 1 | 0 | 0 | 0 | 1 | 0 | 1 | 0 | 1 | 1 | 0 | 0 | 0 | 1 | 1 | 0 | 16 (59%) |
| Retnakaran et al. [[28](#_ENREF_28)] | 1 | 1 | 1 | 0 | 2 | 1 | 1 | 0 | 0 | 1 | 1 | 1 | 0 | 0 | 0 | 1 | 0 | 1 | 0 | 1 | 1 | 1 | 1 | 0 | 1 | 0 | 0 | 17 (63%) |
| Sinkeler et al. [[29](#_ENREF_29)] | 1 | 1 | 1 | 0 | 2 | 1 | 1 | 0 | 0 | 1 | 0 | 0 | 0 | 0 | 1 | 1 | 0 | 1 | 0 | 1 | 0 | 0 | 0 | 0 | 1 | 1 | 0 | 14 (52%) |
| Stoycheff et al. [[30](#_ENREF_30)] | 1 | 1 | 1 | 0 | 2 | 1 | 1 | 0 | 0 | 0 | 0 | 0 | 0 | 0 | 0 | 1 | 0 | 1 | 0 | 1 | 0 | 1 | 1 | 0 | 1 | 1 | 0 | 14 (52%) |
| Takagi et al. [[31](#_ENREF_31)] | 1 | 1 | 1 | 0 | 2 | 1 | 1 | 0 | 0 | 1 | 1 | 1 | 1 | 0 | 0 | 1 | 0 | 1 | 0 | 1 | 1 | 1 | 0 | 0 | 1 | 1 | 0 | 18 (67%) |
| Tanaka et al. [[32](#_ENREF_32)] | 1 | 1 | 1 | 0 | 2 | 1 | 1 | 0 | 0 | 1 | 1 | 1 | 1 | 0 | 0 | 1 | 1 | 1 | 0 | 1 | 1 | 1 | 0 | 0 | 1 | 1 | 0 | 19 (70%) |
| Targher et al. [[33](#_ENREF_33)] | 1 | 1 | 1 | 0 | 2 | 1 | 1 | 0 | 0 | 1 | 1 | 1 | 1 | 0 | 0 | 1 | 1 | 1 | 0 | 1 | 1 | 1 | 0 | 0 | 1 | 1 | 0 | 19 (70%) |
| Unsal et al. [[34](#_ENREF_34)] | 1 | 1 | 1 | 0 | 2 | 1 | 1 | 0 | 1 | 1 | 0 | 0 | 0 | 0 | 0 | 1 | 0 | 1 | 0 | 1 | 1 | 1 | 0 | 0 | 0 | 1 | 0 | 15 (56%) |
| Viana et al. [[35](#_ENREF_35)] | 1 | 1 | 1 | 0 | 2 | 1 | 1 | 0 | 0 | 1 | 0 | 0 | 0 | 0 | 0 | 1 | 0 | 1 | 0 | 1 | 1 | 1 | 0 | 0 | 1 | 1 | 0 | 15 (56%) |
| Vupputuri et al. [[36](#_ENREF_36)] | 1 | 1 | 1 | 0 | 2 | 1 | 1 | 0 | 0 | 1 | 1 | 1 | 1 | 0 | 0 | 1 | 1 | 1 | 0 | 1 | 1 | 1 | 0 | 0 | 0 | 0 | 0 | 17 (63%) |
| Wada et al. [[37](#_ENREF_37)] | 1 | 1 | 1 | 0 | 2 | 1 | 1 | 0 | 0 | 1 | 1 | 1 | 1 | 0 | 0 | 1 | 1 | 1 | 0 | 1 | 1 | 1 | 0 | 0 | 1 | 1 | 0 | 19 (70%) |
| Yang et al. [[38](#_ENREF_38)] | 1 | 1 | 1 | 0 | 2 | 1 | 1 | 0 | 0 | 1 | 1 | 1 | 1 | 0 | 0 | 1 | 1 | 1 | 0 | 1 | 1 | 1 | 0 | 0 | 1 | 1 | 0 | 19 (70%) |
| Yokoyama et al. [[39](#_ENREF_39)] | 1 | 1 | 1 | 0 | 2 | 1 | 1 | 0 | 0 | 1 | 1 | 1 | 0 | 0 | 0 | 1 | 0 | 1 | 0 | 1 | 1 | 1 | 0 | 0 | 0 | 1 | 0 | 16 (59%) |
| Yokoyama et al. [[40](#_ENREF_40)] | 1 | 1 | 1 | 0 | 2 | 1 | 1 | 0 | 0 | 1 | 1 | 1 | 1 | 0 | 0 | 1 | 1 | 1 | 0 | 1 | 1 | 1 | 0 | 0 | 1 | 0 | 0 | 18 (67%) |
| Yokoyama et al. [[41](#_ENREF_41)] | 1 | 1 | 1 | 0 | 2 | 1 | 1 | 0 | 0 | 0 | 1 | 1 | 0 | 0 | 0 | 1 | 1 | 1 | 0 | 1 | 1 | 1 | 0 | 0 | 1 | 0 | 0 | 16 (59%) |
| Zoppini et al. [[42](#_ENREF_42)] | 1 | 1 | 1 | 0 | 2 | 1 | 1 | 0 | 0 | 1 | 1 | 1 | 1 | 0 | 0 | 1 | 1 | 1 | 0 | 1 | 1 | 1 | 0 | 0 | 1 | 1 | 0 | 19 (70%) |
| **^*^**Scored using the Downs and Black quality assessment instrument [[43](#_ENREF_43)] ^¶^Proportional score was calculated by dividing each study’s overall quality assessment score by the sum of points available in the Downs and Black instrument | | | | | | | | | | | | | | | | | | | | | | | | | | | | |

| Additional file: Table 2 Risk estimates for albuminuria/proteinuria measures according to clinical outcomes reported in longitudinal publications | | | | | | | |  |
| --- | --- | --- | --- | --- | --- | --- | --- | --- |
| Author, year |  | Albuminuria/proteinuria  biomarker | | | HR | | 95% CI | |
| Outcome: GFR loss^ǂ^, *n* = 3 | | | | | | | |  |
| De Hauteclocque et al., 2014 [[15](#_ENREF_15)] | | Baseline UACR (log mg/mmol increase) | 1.97 | | | (1.65–2.34)^*^ | | |
| Pavkov et al., 2012 [[26](#_ENREF_26)] | | Baseline ACR doubling | 1.29 | | | (1.11–1.50)^*^ | | |
| Takagi et al., 2015 [[31](#_ENREF_31)] | | Log ACR | | | 2.74 | (1.64–4.57)^*^ | | |
| Outcome: Steep eGFR decline^ǂ^, *n* = 2 | | | | | | | |  |
| De Hauteclocque et al., 2014 [[15](#_ENREF_15)] |  | Baseline UACR (log mg/mmol increase) | | | 1.77 | (1.47–2.13)^*^ | | |
| Kitai et al., 2015 [[19](#_ENREF_19)] |  | Nephrotic range proteinuria | | | 3.89 | 1.08–14.5^*^ | | |
| Outcome: Renal decline^ǂ^ (50% decline in eGFR), *n* = 2 | | | | | | | |  |
| Tanaka et al ., 2015 [[32](#_ENREF_32)] |  | ACR/log10 | | 4.95 | | (4.15–5.90)^*^ | | |
| Wada et al., 2014 [[37](#_ENREF_37)] |  | Normoalbuminuria  Microalbuminuria  Macroalbuminuria | | Reference  3.21  21.86 | | (2.31–4.47)^*^  (16.15–29.59)^*^ | | |
| Outcome: Renal decline^ǂ^ (doubling of serum creatinine), *n* = 3 | | | | | | | |  |
| Lambers Heerspink et al., 2010 [[20](#_ENREF_20)] |  | Per Standard deviation (SD) increment in baseline:  24 hr UAE  24 hr UPE  First morning void UAE  First morning void UACR | | | 3.16  3.02  3.23  4.36 | (2.60–3.86)^*^  (2.53–3.62)^*^  (2.67–3.91)^*^  (3.50–5.45)^*^ | | |
| Monseu et al., 2015 [[21](#_ENREF_21)] |  | Albuminuria (log mg/mmol) | | | 4.23 | (3.07–5.83)^*^ | | |
| Yokoyama et al., 2013 [[41](#_ENREF_41)] |  | Macroalbuminuria remission and ≥ 50% albumin reduction  Macroalbuminuria remission or ≥ 50% albumin reduction | | | 0.30  0.79 | (0.12–0.76)^*^  (0.34–1.82)^*^ | | |
| Outcome: Nephropathy progression^ǂ^, *n* = 2 | | | | | | | |  |
| Chen et al., 2012 [[12](#_ENREF_12)] |  | UACR 30–299.9 mg/gmCr | | | 1.65 | (0.93–2.90) | | |
| Viana et al., 2012 [[35](#_ENREF_35)] |  | UAC ≥ 14 mg/l  ACR ≥ 30 mg/g  UAE ≥ 30 mg/24 h | | | 4.30  4.67  6.76 | (2.22–8.32)^*^  (2.34–9.34)^*^  (3.32–13.77)^*^ | | |
| Outcome: End stage renal disease, *n* = 5 | | | | | | | |  |
| Berhane et al., 2011 [[10](#_ENREF_10)] |  | Increase in albuminuria category | | 2.69 | | (no CI reported) | | |
| de Hauteclocque et al., 2014 [[15](#_ENREF_15)] |  | Baseline UACR (log mg/mmol increase): | | 7.39 | | (3.84–14.20)^*^ | | |
| Elley et al., 2013 [[17](#_ENREF_17)] |  | No albuminuria  Microalbuminuria  Macroalbuminuria  Advanced albuminuria | | Reference  2.01  4.10  11.31 | | (no CI reported)^*^  (no CI reported)^*^  (no CI reported)^*^ | | |
| Pavkov et al., 2012 [[26](#_ENREF_26)] |  | ACR | | 4.87 | | | (3.45–6.88)^*^ | |
| Yang et al., 2006 [[38](#_ENREF_38)] |  | Log10 ACR (mg/mmol) | | | 4.28 | | (2.66–6.88)^*^ | |
| Outcome: Cardiovascular event (excluding cardiovascular mortality), *n* = 5 | | | | | | | |  |
| Chen et al., 2012 [[12](#_ENREF_12)] |  | UACR 30–299.9 mg/gmCr | | | 1.61 | | (0.75–3.44) | |
| Monseu et al., 2015 [[21](#_ENREF_21)] |  | Albuminuria (log mg/mmol):  MACE  Heart failure  Lower limb amputation  PAR | | | 1.33  1.49  1.65  1.49 | | (1.13–1.56)^*^  (1.22–1.82)^*^  (1.21–2.25)^*^  (1.11–2.00)^*^ | |
| Viana et al., 2012 [[35](#_ENREF_35)] |  | UAC ≥ 14 mg/l  ACR ≥ 30 mg/g  UAE ≥ 30 mg/24-h | | | 3.25  2.89  2.20 | | (1.43–7.38)^*^  (1.29–6.45)^*^  (2.08–2.49)^*^ | |
| Wada et al., 2014 [[37](#_ENREF_37)] |  | Normoalbuminuria  Microalbuminuria  Macroalbuminuria | | | Reference  1.38  2.05 | | (1.14–1.67)^*^  (1.61–2.58)^*^ | |
| Yokoyama et al., 2012 [[40](#_ENREF_40)] |  | Albuminuria progression | | | 10.00 | | (1.58–63.23)^*^ | |
| Outcome: Cardiovascular mortality, *n* = 4 | | | | | | | |  |
| Bruno et al., 2007 [[11](#_ENREF_11)] |  | AER: < 20 μg/min  AER 20–200  AER > 200 | | | Reference  1.06  2.00 | | (0.80–1.40)  (1.48–2.71)^*^ | |
| Cox et al., 2013 [[13](#_ENREF_13)] |  | UACR | | | 1.47 | | (1.24–1.74)^*^ | |
| Monseu et al., 2015 [[21](#_ENREF_21)] |  | Albuminuria (log mg/mmol) | | | 1.46 | | (1.20–1.77)^*^ | |
| Targher et al., 2011 [[33](#_ENREF_33)] |  | Albuminuria/1 SD increment  Microalbuminuria  Macroalbuminuria | | | 1.19  1.56  3.40 | | (1.01–1.40)^*^  (0.85–3.10)  (1.50–7.80)^*^ | |
| Outcome: All-cause mortality, *n* = 10 | | | | | | | |  |
| Berhane et al., 2011 [[10](#_ENREF_10)] |  | Increase in albuminuria category | | | 1.37 | (no CI reported) | | |
| Bruno et al., 2007 [[11](#_ENREF_11)] |  | AER: < 20 μg/min  AER 20–200  AER > 200 | | | Reference  1.30  1.91 | (1.08–1.57)^*^  (1.54–2.38)^*^ | | |
| Cox et al., 2013 [[13](#_ENREF_13)] |  | UACR | | | 1.35 | (1.19–1.53)^*^ | | |
| Chen et al., 2012 [[12](#_ENREF_12)] |  | UACR 30–299.9 mg/gmCr | | | 1.80 | (0.71–4.56) | | |
| Monseu et al., 2015 [[21](#_ENREF_21)] |  | Albuminuria (log mg/mmol) | | | 1.38 | (1.19–1.59)^*^ | | |
| Murussi et al., 2007 [[23](#_ENREF_23)] |  | Higher UAE levels (> 5μg/min) | | | 2.70 | (1.20–6.10)^*^ | | |
| Tanaka et al., 2015 [[32](#_ENREF_32)] |  | ACR/log10, SD | | | 1.31 | (0.99–1.72) | | |
| Targher et al., 2011 [[33](#_ENREF_33)] |  | Albuminuria/1 SD increment: | | | 1.14 | (1.01–1.30)^*^ | | |
| Viana et al., 2012 [[35](#_ENREF_35)] |  | UAC ≥ 14 mg/l  ACR ≥ 30 mg/g  UAE ≥ 30 mg/24-h | | | 5.51  5.07  2.47 | (1.16–26.22)^*^  (1.01–24.88)^*^  (0.72–8.42) | | |
| Wada et al., 2014 [[37](#_ENREF_37)] |  | Normoalbuminuria  Microalbuminuria  Macroalbuminuria | | | Reference  1.37  3.60 | (0.99–1.89)  (2.53–5.20)^*^ | | |
| ^*^Hazard ratio is significantly different from 1.0  ^ǂ^Renal decline was measured in multiple ways in the included studies  *ACR* albumin-to-creatinine ratio, *AER* albumin excretion rate, *CI* confidence interval, *Cr* creatinine, *GFR* glomerular filtration rate, *HR* hazard ratio, *MACE* major adverse coronary event, *PAR* peripheral artery revascularization, *RRT* renal replacement therapy, *SD* standard deviation, *UAC* urinary albumin concentration, *UACR* urinary albumin-to-creatinine ratio, *UAE* urinary albumin excretion, *UPE* urinary protein excretion | | | | | | | |  |

| Additional file: Table 3 Risk estimates for serum creatinine, uric acid, and estimated glomerular filtration rate measures according to clinical outcomes reported in longitudinal publications | | | | | | | | | | |  |
| --- | --- | --- | --- | --- | --- | --- | --- | --- | --- | --- | --- |
| Author, year |  | | Serum creatinine/uric acid/eGFR  biomarker | HR | 95% CI | | | | | |  |
| Outcome: Renal decline^ǂ^ (50% decline in eGFR), *n* = 2 | | | |  | |  |  |  | | |  |
| Takagi et al., 2015 [[31](#_ENREF_31)] |  | eGFR | | 0.89 | (0.87–0.90)^*^ | | | | | |  |
| Tanaka et al., 2015 [[32](#_ENREF_32)] |  | eGFR/SD | | 0.88 | (0.76–1.03) | | | | | |  |
| Outcome: Renal decline^ǂ^ (doubling of serum creatinine), *n* = 6 | | | | | | | | | | |  |
| Araki et al., 2012 [[7](#_ENREF_7)] |  | sUA < 4.5 (mg/dl)  sUA 4.5–6.5 (mg/dl)  sUA ≥ 6.5 (mg/dl) | | Reference  3.42  8.65 | (0.94–12.43)  (2.60–28.78)^*^ | | | | | |  |
| Lambers Heerspink et al., 2010 [[20](#_ENREF_20)] |  | Per SD decrement in baseline:  Urinary creatinine (g/L) – FMV  24 hr UCC (g/L)  24 hr UCE (g/24 hr) | | 1.38  1.52  1.82 | (1.11–1.72)^*^  (1.30–1.78)^*^  (1.37–2.42)^*^ | | | | | |  |
| Monseu et al., 2015 [[21](#_ENREF_21)] |  | eGFR (10 mL/min/1.73m^2^) | | 0.78 | (0.70–0.87)^*^ | | | | | |  |
| Bentata et al., 2014 [[9](#_ENREF_9)] |  | Baseline eGFR | | 5.67 | (1.23–9.77)^*^ | | | | | |  |
| Chen et al., 2012 [[12](#_ENREF_12)] |  | eGFR 30–59.9 | | 1.13 | (0.62–2.04) | | | | | |  |
| Murussi et al., 2007 [[23](#_ENREF_23)] |  | Lower GFR | | 0.98 | (0.97–1.00) | | | | | |  |
| Outcome: Creatinine clearance^ǂ^, *n* = 1 | | | | | | | | | | |  |
| Retnakaran et al., 2006 [[28](#_ENREF_28)] | | Plasma creatinine (10 umol/l) | | 1.34 | (1.28–1.40)^*^ | | | | | |  |
| Outcome: Nephropathy progression^ǂ^, *n* = 1 | | | | | | | | | | |  |
| Azubike et al., 2013 [[8](#_ENREF_8)] |  | Baseline eGFR: | | 5.67 | (1.23–9.77)^*^ | | | | | |  |
| Outcome: End stage renal disease, *n* = 5 | | | | | | | | | | |  |
| Berhane et al., 2011 [[10](#_ENREF_10)] |  | 10 ml/min/1.73m^2^ lower eGFR | | 1.36 | (no CI reported) | | | | | |  |
| de Hauteclocque et al., 2014 [[15](#_ENREF_15)] |  | Baseline eGFR < 60  Steep eGFR decline | | 12.31  3.31 | (3.60–42.04)^*^  (1.38–7.09)^*^ | | | | | |  |
| Elley et al., 2013 [[17](#_ENREF_17)] |  | eGFR | | 0.96 | (no CI reported)^*^ | | | | | |  |
| Pavkov et al., 2012 [[26](#_ENREF_26)] |  | GFR slope | | 4.46 | (3.11–6.41)^*^ | | | | | |  |
| Yang et al., 2006 [[38](#_ENREF_38)] |  | eGFR (10 ml/min/1.73 m^2^) ^41^ | | 0.73 | (0.61–0.88)^*^ | | | | | |  |
| Outcome: Cardiovascular event (excluding cardiovascular mortality), *n* = 3 | | | | | | | | |  |  |  |
| Chen et al., 2012 [[12](#_ENREF_12)] |  | eGFR 30–59.9 | | 0.97 | (0.44–2.15) | | | | | |  |
| Monseu et al., 2015 [[21](#_ENREF_21)] |  | eGFR (10 mL/min/1.73m^2^) | | 0.89 | (0.84–0.94)^*^ | | | | | |  |
| Yokoyama et al., 2012 [[40](#_ENREF_40)] |  | eGFR progression | | 1.27 | (0.24–6.59) | | | | | |  |
| Outcome: Cardiovascular mortality, *n* = 5 | | | | | | | | | | |  |
| Bruno et al., 2007 [[11](#_ENREF_11)] |  | Serum creatinine:  < 80 mmol/l  80–87  88–103  > 103  eGFR:  eGFR ≥ 90  eGFR 60–89  eGFR 45–59  eGFR 30–44  eGFR 15–29 | | Reference  0.94  1.03  1.05  Reference  0.65  0.79  0.67  2.03 | (0.61–1.45)  (0.76–1.38)  (1.08–1.98)^*^  (0.39–1.11)  (0.45–1.39)  (0.35–1.27)  (0.85–4.85) | | | | | |  |
| Cox et al., 2013 [[13](#_ENREF_13)] |  | Serum creatinine  eGFR | | 1.49  0.68 | (1.15–1.92)^*^  (0.53–0.89)^*^ | | | | | |  |
| Monseu et al., 2015 [[21](#_ENREF_21)] |  | eGFR (/10 mL/min/1.73m^2^) | | 0.87 | (0.81–0.94)^*^ | | | | | |  |
| Sinkeler et al., 2013 [[29](#_ENREF_29)] |  | Cr excretion rate | | 0.49 | (0.33–0.73)^*^ | | | | | |  |
| Targher et al., 2011 [[33](#_ENREF_33)] |  | eGFR/1 SD decrement  eGFR ≥ 90  eGFR 60–89  eGFR 30–59  eGFR < 30 | | 1.51  Reference  0.98  1.16  6.81 | (1.05–2.20)^*^  (0.44–2.80)  (0.45–3.70)  (1.7–27.10)^*^ | | | | | |  |
| Outcome: All-cause mortality, *n* = 8 | | | | | | | | | | | |
| Berhane et al., 2011 [[10](#_ENREF_10)] |  | 10 ml/min/1.73m^2^ lower eGFR: | | 1.15 | (no CI reported) | | | | | |  |
| Bruno et al., 2007 [[11](#_ENREF_11)] |  | Serum creatinine:  < 80 moll/l  80–87  88–103  > 103  eGFR:  eGFR ≥ 90  eGFR 60–89  eGFR 45–59  eGFR 30–44  eGFR 15–29 | | Reference  0.98  1.03  1.23  Reference  0.73  0.93  0.74  2.36 | (0.73–1.31)  (0.84–1.27)  (0.99–1.53)  (0.51–1.05)  (0.62–1.37)  (0.47–1.16)  (1.28–4.34)^*^ | | | | | |  |
| Chen et al., 2012 [[12](#_ENREF_12)] |  | eGFR 30–59.9 | | 0.87 | (0.35–2.20) | | | | | |  |
| Cox et al., 2013 [[13](#_ENREF_13)] |  | Serum creatinine  eGFR | | 1.29  0.77 | (1.09–1.52)^*^  (0.65–0.91)^*^ | | | | | |  |
| Monseu et al., 2015 [[21](#_ENREF_21)] |  | eGFR (10 mL/min/1.73m^2^) | | 0.92 | (0.87–0.97)^*^ | | | | | |  |
| Sinkeler et al., 2013 [[29](#_ENREF_29)] |  | Doubling of creatinine excretion rate | | 0.39 | (0.29–0.52)^*^ | | | | | |  |
| Tanaka et al., 2015 [[32](#_ENREF_32)] |  | eGFR/SD | | 0.58 | (0.42–0.81)^*^ | | | | | |  |
| Targher et al., 2011 [[33](#_ENREF_33)] |  | eGFR/SD decrement | | 1.53 | (1.20–2.00)^*^ | | | | | |  |
| ^*^Hazard ratio is significantly different from 1.0 ^ǂ^Renal decline was measured in multiple ways in the included studies. Only studies in which decline was measured by 50% eGFR or by a doubling of serum creatinine are presented here *CI* confidence interval, *eGFR* estimated glomerular filtration rate (units in mL/min/1.73m^2^ unless otherwise specified), *FMV* first morning void, *GFR* glomerular filtration rate, *HR* hazard ratio, *RRT* renal replacement therapy, *SD* standard deviation, *sUA* serum uric acid, *UCC* urinary creatinine concentration, *UCE* urinary creatinine excretion | | | | | | | | | | |  |

| Additional file: Table 4 Risk estimates for combined albuminuria/proteinuria and serum creatinine/uric acid/estimated glomerular filtration rate measures according to clinical outcomes reported in longitudinal publications | | | | | | | | | | |
| --- | --- | --- | --- | --- | --- | --- | --- | --- | --- | --- |
| Author, year |  | | | | Albuminuria/proteinuria and serum creatinine/uric acid/eGFR  biomarkers | HR/RR | | 95% CI | | |
| Outcome: Renal decline^ǂ^ (50% decline in eGFR), *n* = 1 | | | | | | | | | | |
| Wada et al., 2014 [[37](#_ENREF_37)] |  | | Normoalbuminuria and eGFR > 60  Normoalbuminuria and eGFR < 30  Microalbuminuria and eGFR > 60  Macroalbuminuria and eGFR > 60  Macroalbuminuria and eGFR 30–59 | | | Reference  49.82  3.26  13.60  33.00 | | (29.90–83.00)^*^  (2.34–4.55)^*^  (9.30–20.00)^*^  (22.70–48.20)^*^ | | |
| Outcome: Nephropathy progression^ǂ^, *n* = 2 | | | | | | | | | | |
| De Cosmo et al., 2015 [[14](#_ENREF_14)] |  | | Normoalbuminuria + eGFR < 60  sUA by quintiles:  Relative risk for 4 year outcomes Q1 Q2 Q3 Q4 Q5  Albuminuria + eGFR < 60:  sUA by quintiles:  Relative risk for 4 year outcomes Q1  Q2 Q3 Q4 Q5 | | | Reference  1.46  1.44  1.95  2.61  Reference  0.99  1.20  1.08  1.54 | | (1.14–1.88)^*^  (1.11–1.87)^*^  (1.48–2.58)^*^  (1.98–3.42)^*^  (0.69–1.42)  (0.80–1.80)  (0.76–1.52)  (1.13–2.09) | | |
| Vupputuri et al., 2011 [[36](#_ENREF_36)] |  | | Normoalbuminuria + eGFR (10 ml/min/1.73m^2^)  Microalbuminuria + eGFR (10 ml/min/1.73m^2^)  Macroalbuminuria + eGFR (10 ml/min/1.73m^2^) | | | 1.06  0.99  0.34 | | (1.03–1.08)^*^  (0.94–1.04)  (0.26–0.44)^*^ | | |
| Outcome: End stage renal disease, *n* = 2 | | | | | | | | | | |
| Berhane et al., 2011 [[10](#_ENREF_10)] |  | | Normoalbuminuria + eGFR ≥ 120  Microalbuminuria + eGFR ≥ 120  Macroalbuminuria + eGFR ≥ 120  Microalbuminuria + eGFR 90–119  Macroalbuminuria + eGFR 90–119  Normoalbuminuria + eGFR 60–89  Microalbuminuria + eGFR 60–89  Macroalbuminuria + eGFR 60–89  Normoalbuminuria + eGFR 30–59  Microalbuminuria + eGFR 30–59  Macroalbuminuria + eGFR 30–59  Macroalbuminuria + eGFR 15–29 | | | 1.20  1.30  6.90  3.10  8.70  1.50  6.00  14.60  5.70  3.80  56.60  290.90 | | (0.80–2.00)  (0.70–2.40)  (3.60–13.10)^*^  (2.00–4.80)^*^  (5.60–13.60)^*^  (0.60–4.00)  (3.00–11.00)^*^  (8.70–24.40)^*^  (0.80–42.10)  (0.50–28.10)  (31.80–100.70)^*^  (130.00–650.80)^*^ | | |
| Packham et al., 2012 [[24](#_ENREF_24)] |  | | ACR < 2.0 and eGFR ≤ 30  ACR < 2.0 and eGFR 30–45  ACR < 2.0 and eGFR > 45  ACR 1.0–2.0 and eGFR ≤ 30  ACR 1.0–2.0 and eGFR 30–45  ACR 1.0–2.0 and eGFR > 45  ACR ≤ 1.0 and eGFR ≤ 30  ACR ≤ 1.0 and eGFR 30–45  ACR ≤ 1.0 and eGFR > 45 | | | 12.87  7.46  7.40  7.12  3.47  2.80  3.61  1.49  Reference | | (5.97–27.74)^*^  (3.63–15.33)^*^  (3.32–16.47)^*^  (3.16–16.04)^*^  (1.63–7.40)^*^  (1.18–6.64)^*^  (1.49–8.73)^*^  (0.64–3.48) | | |
| Outcome: Cardiovascular event (excluding cardiovascular mortality), *n* = 2 | | | | | | | | | | |
| Wada et al., 2014 [[37](#_ENREF_37)] |  | | Normoalbuminuria and eGFR > 60  Normoalbuminuria and eGFR < 30  Microalbuminuria and eGFR > 60  Macroalbuminuria and eGFR > 60  Macroalbuminuria and eGFR 30–59 | | | Reference  1.54  1.40  1.90  2.09 | | (1.00–2.39)^*^  (1.16–1.69)^*^  (1.36–2.65)^*^  (1.54–2.84)^*^ | | |
| Yokoyama et al., 2012 [[40](#_ENREF_40)] | |  | | ACR < 3.5 and eGFR ≥ 90  ACR < 3.5 and eGFR 60–89  ACR < 3.5 and eGFR < 60  ACR 3.5–34.9 and eGFR ≥ 90  ACR 3.5–34.9 and eGFR 60–89  ACR 3.5–34.9 and eGFR < 60  ACR > 35 and eGFR ≥ 90  ACR > 35 and eGFR 60–89  ACR > 35 and eGFR < 60 | | | Reference  1.43  1.40  1.65  1.78  2.16  1.80  4.66  5.07 | | (0.66–3.12)  (0.48–4.13)  (0.54–5.07)  (0.74–4.35)  (0.72–6.46)  (0.22–14.42)  (1.63–13.35)^*^  (1.69–15.21)^*^ | |
| Outcome: All-cause mortality, *n* = 3 | | | | | | | | | | |
| Berhane et al., 2011 [[10](#_ENREF_10)] |  | | Normoalbuminuria and eGFR 90–119  Normoalbuminuria and eGFR ≥ 120  Microalbuminuria and eGFR ≥ 120  Macroalbuminuria and eGFR ≥ 120  Microalbuminuria and eGFR 90–119  Macroalbuminuria and eGFR 90–119  Normoalbuminuria and eGFR 60–89  Microalbuminuria and eGFR 60–89  Macroalbuminuria and eGFR 60–89  Normoalbuminuria and eGFR 30–59  Microalbuminuria and eGFR 30–59  Macroalbuminuria and eGFR 30–59  Macroalbuminuria and eGFR 15–29 | | | | Reference  1.20  1.00  2.90  1.50  2.00  1.60  2.00  2.50  2.30  4.30  6.50  7.50 | | | (0.90–1.70)  (0.60–1.70)  (1.70–5.10)^*^  (1.10–1.90)^*^  (1.40–2.70)^*^  (1.10–2.30)^*^  (1.30–3.00)^*^  (1.80–3.60)^*^  (0.80–6.10)  (2.10–8.90)^*^  (4.40–9.80)^*^  (4.20–13.50)^*^ |
| Wada et al., 2014 [[37](#_ENREF_37)] |  | | Normoalbuminuria and eGFR > 60  Normoalbuminuria and eGFR < 30  Microalbuminuria and eGFR > 60  Macroalbuminuria and eGFR > 60  Macroalbuminuria and eGFR 30–59 | | | Reference  7.08  1.30  2.34  4.59 | | (4.16–12.05)^*^  (0.93–1.81)  (1.35–4.04)^*^  (2.90–7.25)^*^ | | |
| Yokoyama et al., 2012 [[40](#_ENREF_40)] |  | | ACR < 3.5 and eGFR ≥ 90  ACR < 3.5 and eGFR 60–89  ACR < 3.5 and eGFR < 60  ACR 3.5–34.9 and eGFR ≥ 90  ACR 3.5–34.9 and eGFR 60–89  ACR 3.5–34.9 and eGFR < 60  ACR > 35 and eGFR ≥ 90  ACR > 35 and eGFR 60–89  ACR > 35 and eGFR < 60 | | | Reference  0.96  0.88  1.44  1.18  1.17  1.97  3.26  3.89 | | (0.53–1.73)  (0.35–2.20)  (0.61–3.40)  (0.58–2.39)  (0.45–3.10)  (0.45–6.68)  (1.38–1.39)^*^  (1.51–9.02)^*^ | | |
| ^*^Hazard ratio is significantly different from 1.0 ^ǂ^Renal decline was measured in multiple ways in the included studies *ACR* albumin-to-creatinine ratio, *CI* confidence interval, *eGFR* estimated glomerular filtration rate (units in mL/min/1.73m^2^ unless otherwise specified), *GFR* glomerular filtration rate, *HR* hazard ratio, *RR* relative risk, *SD* standard deviation, *sUA* serum uric acid | | | | | | | | | | |

**Additional files: References**

1. Afghahi H, Cederholm J, Eliasson B, Zethelius B, Gudbjornsdottir S, Hadimeri H, Svensson MK. Risk factors for the development of albuminuria and renal impairment in type 2 diabetes--the Swedish National Diabetes Register (NDR). Nephrol Dial Transplant. 2011;26(4):1236-43.
2. Afkarian M, Sachs MC, Kestenbaum B, Hirsch IB, Tuttle KR, Himmelfarb J, de Boer IH. Kidney disease and increased mortality risk in type 2 diabetes. J Am Soc Nephrol. 2013;24(2):302-8.
3. Al Suleiman MH, Kfoury HK, Jondeby MS, Burgos NS, Al Hayyan H, Al Sayyari A. Progression of Diabetic Nephropathy in Saudi Patients With Type 2 Diabetes Mellitus. The Endocrinologist. 2008;18(5):230-2.
4. Altemtam N, Russell J, El Nahas M. A study of the natural history of diabetic kidney disease (DKD). Nephrol Dial Transplant. 2012;27(5):1847-54.
5. Alwakeel JS, Isnani AC, Alsuwaida A, Alharbi A, Shaffi SA, Almohaya S, Al Ghonaim M. Factors affecting the progression of diabetic nephropathy and its complications: a single-center experience in Saudi Arabia. Ann Saudi Med. 2011;31(3):236-42.
6. Andresdottir G, Jensen ML, Carstensen B, Parving HH, Rossing K, Hansen TW, Rossing P. Improved survival and renal prognosis of patients with type 2 diabetes and nephropathy with improved control of risk factors. Diabetes Care. 2014;37(6):1660-7.
7. Araki S, Nishio Y, Araki A, Umegaki H, Sakurai T, Iimuro S, Ohashi Y, Uzu T, Maegawa H, Kashiwagi A et al. Factors associated with progression of diabetic nephropathy in Japanese elderly patients with type 2 diabetes: sub-analysis of the Japanese Elderly Diabetes Intervention Trial. Geriatr Gerontol Int. 2012;12 Suppl 1:127-33.
8. Azubike C.O., Unuigbe E.I. Progression of diabetic nephropathy: A twelve-year follow-up of type2 diabetic patients. Journal of Medicine and Biomedical Research. 2013;12(1):105-15.
9. Bentata Y, Abouqal R. Does albuminuria predict renal risk and/or cardiovascular risk in obese type 2 diabetic patients? Am J Cardiovasc Dis. 2014;4(1):26-30.
10. Berhane AM, Weil EJ, Knowler WC, Nelson RG, Hanson RL. Albuminuria and estimated glomerular filtration rate as predictors of diabetic end-stage renal disease and death. Clin J Am Soc Nephrol. 2011;6(10):2444-51.
11. Bruno G, Merletti F, Bargero G, Novelli G, Melis D, Soddu A, Perotto M, Pagano G, Cavallo-Perin P. Estimated glomerular filtration rate, albuminuria and mortality in type 2 diabetes: the Casale Monferrato study. Diabetologia. 2007;50(5):941-8.
12. Chen YH, Chen HS, Tarng DC. More impact of microalbuminuria on retinopathy than moderately reduced GFR among type 2 diabetic patients. Diabetes Care. 2012;35(4):803-8.
13. Cox AJ, Hsu FC, Carr JJ, Freedman BI, Bowden DW. Glomerular filtration rate and albuminuria predict mortality independently from coronary artery calcified plaque in the Diabetes Heart Study. Cardiovasc Diabetol. 2013;12:68.
14. De Cosmo S, Viazzi F, Pacilli A, Giorda C, Ceriello A, Gentile S, Russo G, Rossi MC, Nicolucci A, Guida P et al. Serum Uric Acid and Risk of CKD in Type 2 Diabetes. Clin J Am Soc Nephrol. 2015;10(11):1921-9.
15. de Hauteclocque A, Ragot S, Slaoui Y, Gand E, Miot A, Sosner P, Halimi JM, Zaoui P, Rigalleau V, Roussel R et al. The influence of sex on renal function decline in people with Type 2 diabetes. Diabet Med. 2014;31(9):1121-8.
16. Dunkler D, Gao P, Lee SF, Heinze G, Clase CM, Tobe S, Teo KK, Gerstein H, Mann JF, Oberbauer R. Risk Prediction for Early CKD in Type 2 Diabetes. Clin J Am Soc Nephrol. 2015;10(8):1371-9.
17. Elley CR, Robinson T, Moyes SA, Kenealy T, Collins J, Robinson E, Orr-Walker B, Drury PL. Derivation and validation of a renal risk score for people with type 2 diabetes. Diabetes Care. 2013;36(10):3113-20.
18. Jardine MJ, Hata J, Woodward M, Perkovic V, Ninomiya T, Arima H, Zoungas S, Cass A, Patel A, Marre M et al. Prediction of kidney-related outcomes in patients with type 2 diabetes. Am J Kidney Dis. 2012;60(5):770-8.
19. Kitai Y, Doi Y, Osaki K, Sugioka S, Koshikawa M, Sugawara A. Nephrotic range proteinuria as a strong risk factor for rapid renal function decline during pre-dialysis phase in type 2 diabetic patients with severely impaired renal function. Clin Exp Nephrol. 2015;19(6):1037-43.
20. Lambers Heerspink HJ, Gansevoort RT, Brenner BM, Cooper ME, Parving HH, Shahinfar S, de Zeeuw D. Comparison of different measures of urinary protein excretion for prediction of renal events. J Am Soc Nephrol. 2010;21(8):1355-60.
21. Monseu M, Gand E, Saulnier PJ, Ragot S, Piguel X, Zaoui P, Rigalleau V, Marechaud R, Roussel R, Hadjadj S et al. Acute Kidney Injury Predicts Major Adverse Outcomes in Diabetes: Synergic Impact With Low Glomerular Filtration Rate and Albuminuria. Diabetes Care. 2015;38(12):2333-40.
22. Moriya T, Tsuchiya A, Okizaki S, Hayashi A, Tanaka K, Shichiri M. Glomerular hyperfiltration and increased glomerular filtration surface are associated with renal function decline in normo- and microalbuminuric type 2 diabetes. Kidney Int. 2012;81(5):486-93.
23. Murussi M, Campagnolo N, Beck MO, Gross JL, Silveiro SP. High-normal levels of albuminuria predict the development of micro- and macroalbuminuria and increased mortality in Brazilian Type 2 diabetic patients: an 8-year follow-up study. Diabet Med. 2007;24(10):1136-42.
24. Packham DK, Alves TP, Dwyer JP, Atkins R, de Zeeuw D, Cooper M, Shahinfar S, Lewis JB, Lambers Heerspink HJ. Relative incidence of ESRD versus cardiovascular mortality in proteinuric type 2 diabetes and nephropathy: results from the DIAMETRIC (Diabetes Mellitus Treatment for Renal Insufficiency Consortium) database. Am J Kidney Dis. 2012;59(1):75-83.
25. Pavkov ME, Knowler WC, Hanson RL, Bennett PH, Nelson RG. Predictive power of sequential measures of albuminuria for progression to ESRD or death in Pima Indians with type 2 diabetes. Am J Kidney Dis. 2008;51(5):759-66.
26. Pavkov ME, Knowler WC, Lemley KV, Mason CC, Myers BD, Nelson RG. Early renal function decline in type 2 diabetes. Clin J Am Soc Nephrol. 2012;7(1):78-84.
27. Pavkov ME, Knowler WC, Hanson RL, Williams DE, Lemley KV, Myers BD, Nelson RG. Comparison of serum cystatin C, serum creatinine, measured GFR, and estimated GFR to assess the risk of kidney failure in American Indians with diabetic nephropathy. Am J Kidney Dis. 2013;62(1):33-41.
28. Retnakaran R, Cull CA, Thorne KI, Adler AI, Holman RR. Risk factors for renal dysfunction in type 2 diabetes: U.K. Prospective Diabetes Study 74. Diabetes. 2006;55(6):1832-9.
29. Sinkeler SJ, Kwakernaak AJ, Bakker SJ, Shahinfar S, Esmatjes E, de Zeeuw D, Navis G, Lambers Heerspink HJ. Creatinine excretion rate and mortality in type 2 diabetes and nephropathy. Diabetes Care. 2013;36(6):1489-94.
30. Stoycheff N, Stevens LA, Schmid CH, Tighiouart H, Lewis J, Atkins RC, Levey AS. Nephrotic syndrome in diabetic kidney disease: an evaluation and update of the definition. Am J Kidney Dis. 2009;54(5):840-9.
31. Takagi M, Babazono T, Uchigata Y. Differences in risk factors for the onset of albuminuria and decrease in glomerular filtration rate in people with Type 2 diabetes mellitus: implications for the pathogenesis of diabetic kidney disease. Diabet Med. 2015;32(10):1354-60.
32. Tanaka N, Babazono T, Takagi M, Yoshida N, Toya K, Nyumura I, Hanai K, Uchigata Y. Albuminuria and reduced glomerular filtration rate for predicting the renal outcomes in type 2 diabetic patients. Nephrology (Carlton). 2015;20(8):531-8.
33. Targher G, Zoppini G, Chonchol M, Negri C, Stoico V, Perrone F, Muggeo M, Bonora E. Glomerular filtration rate, albuminuria and risk of cardiovascular and all-cause mortality in type 2 diabetic individuals. Nutr Metab Cardiovasc Dis. 2011;21(4):294-301.
34. Unsal A, Koc Y, Basturk T, Akgun AO, Sakaci T, Ahbap E. Risk factors for progression of renal disease in patient with diabetic nephropathy. Eur Rev Med Pharmacol Sci. 2012;16(7):878-83.
35. Viana LV, Gross JL, Camargo JL, Zelmanovitz T, da Costa Rocha EP, Azevedo MJ. Prediction of cardiovascular events, diabetic nephropathy, and mortality by albumin concentration in a spot urine sample in patients with type 2 diabetes. J Diabetes Complications. 2012;26(5):407-12.
36. Vupputuri S, Nichols GA, Lau H, Joski P, Thorp ML. Risk of progression of nephropathy in a population-based sample with type 2 diabetes. Diabetes Res Clin Pract. 2011;91(2):246-52.
37. Wada T, Haneda M, Furuichi K, Babazono T, Yokoyama H, Iseki K, Araki S, Ninomiya T, Hara S, Suzuki Y et al. Clinical impact of albuminuria and glomerular filtration rate on renal and cardiovascular events, and all-cause mortality in Japanese patients with type 2 diabetes. Clin Exp Nephrol. 2014;18(4):613-20.
38. Yang XL, So WY, Kong AP, Clarke P, Ho CS, Lam CW, Ng MH, Lyu RR, Yin DD, Chow CC et al. End-stage renal disease risk equations for Hong Kong Chinese patients with type 2 diabetes: Hong Kong Diabetes Registry. Diabetologia. 2006;49(10):2299-308.
39. Yokoyama H, Kanno S, Takahashi S, Yamada D, Honjo J, Saito K, Sone H, Haneda M. Risks for glomerular filtration rate decline in association with progression of albuminuria in type 2 diabetes. Nephrol Dial Transplant. 2011;26(9):2924-30.
40. Yokoyama H, Araki S, Haneda M, Matsushima M, Kawai K, Hirao K, Oishi M, Sugimoto K, Sone H, Maegawa H et al. Chronic kidney disease categories and renal-cardiovascular outcomes in type 2 diabetes without prevalent cardiovascular disease: a prospective cohort study (JDDM25). Diabetologia. 2012;55(7):1911-8.
41. Yokoyama H, Araki S, Honjo J, Okizaki S, Yamada D, Shudo R, Shimizu H, Sone H, Moriya T, Haneda M. Association between remission of macroalbuminuria and preservation of renal function in patients with type 2 diabetes with overt proteinuria. Diabetes Care. 2013;36(10):3227-33.
42. Zoppini G, Targher G, Chonchol M, Ortalda V, Negri C, Stoico V, Bonora E. Predictors of estimated GFR decline in patients with type 2 diabetes and preserved kidney function. Clin J Am Soc Nephrol. 2012;7(3):401-8.
43. Downs SH, Black N. The feasibility of creating a checklist for the assessment of the methodological quality both of randomised and non-randomised studies of health care interventions. J Epidemiol Community Health. 1998;52(6):377-84.
